# Validating an education program for families of persons with dementia in India

**DOI:** 10.1002/alz70858_107815

**Published:** 2025-12-26

**Authors:** Kshama Bangera, Sebestina Anita Dsouza

**Affiliations:** ^1^ Manipal Academy of Higher Education, Manipal, Karnataka, India

## Abstract

**Background:**

In resource constrained settings and collective cultures such as India, family is the primary and major source of care for a person with dementia. It is important to educate the family unit about dementia. In the preliminary study, we developed an education program for families of persons with dementia. The novel features of the education program were its family centered approach, case‐based approach and use of adult learning principles. The present study aimed to establish the content validity of the education program.

**Method:**

Fourteen stakeholders including health care professionals and family caregivers were invited as experts to validate the education program. The survey questionnaire included a 5‐point Likert scale to rate the program's content, relevance and feasibility. The survey also included open‐ended questions to elicit feedback and suggestions. The content validity index (CVI) was computed for individual items and total score. Thematic analysis was carried out on the qualitative data.

**Result:**

Consensus was attained on all items and total score (I‐CVI > 0.78), except for number (I‐CVI 0.61) and duration (I‐CVI 0.53) of sessions. Experts appreciated the program's case‐based and participatory approach but raised concerns about the delivery and accessibility for lower‐educated and rural populations. Based on the expert suggestions the program was reorganized to address the delivery related concerns.

**Conclusion:**

This study established the content validity of the education program. The education program for families of persons with dementia is a promising, contextually relevant and feasible intervention to support primary caregivers and family members cope with caregiving demands inherent in dementia care. The program has scope for application in diverse settings especially resource‐constrained low‐and‐middle income countries and collective cultures. Further research is required to assess its application in practice and accessibility to diverse population in the Indian context

